# Epidemiological and Molecular Characterization of Dengue Virus Circulating in Bhutan, 2013-2014

**DOI:** 10.1371/journal.pntd.0004010

**Published:** 2015-08-21

**Authors:** Sangay Zangmo, Chonticha Klungthong, Piyawan Chinnawirotpisan, Srisurang Tantimavanich, Nathamon Kosoltanapiwat, Butsaya Thaisomboonsuk, Kelzang Phuntsho, Sonam Wangchuk, In-Kyu Yoon, Stefan Fernandez

**Affiliations:** 1 Faculty of Medical Technology, Mahidol University, Bangkok, Thailand; 2 Public Health Laboratory, Ministry of Health, Thimphu, Bhutan; 3 Department of Virology, Armed Force Research Institute of Medical Sciences, Bangkok, Thailand; 4 Faculty of Tropical Medicine, Mahidol University, Bangkok, Thailand; Duke-NUS Graduate Medical School, SINGAPORE

## Abstract

Dengue is one of the most significant public health problems in tropical and subtropical countries, and is increasingly being detected in traditionally non-endemic areas. In Bhutan, dengue virus (DENV) has only recently been detected and limited information is available. In this study, we analyzed the epidemiological and molecular characteristics of DENV in two southern districts in Bhutan from 2013–2014. During this period, 379 patients were clinically diagnosed with suspected dengue, of whom 119 (31.4%) were positive for DENV infection by NS1 ELISA and/or nested RT-PCR. DENV serotypes 1, 2 and 3 were detected with DENV-1 being predominant. Phylogenetic analysis of DENV-1 using envelope gene demonstrated genotype V, closely related to strains from northern India.

## Author Summary

We describe the epidemiological and molecular features of DENV currently circulating in the two southwestern districts of Bhutan, demonstrating a shift in serotype dominance from previous DENV-3 (2004–2006) to current DENV-1 (2013–2014). The presence of the dengue virus in Bhutan is a relatively recent one. Unfortunately, dengue epidemiological and molecular data in this country is scarce. A fever outbreak in 2013 and 2014 saw patients seeking care at medical facilities in two district of southwestern Bhutan bordering with India. Analyses of serum specimens collected from these patients indicated that dengue virus was at least a major source of this outbreak. These specimens were analyzed in the Public Health Laboratory in Bhutan and in AFRIMS, Thailand. With a combination of three different assays, we established that 31% of all cases captured were caused by dengue virus, although the proportion was higher in 2013 than in 2014. Three different serotypes of dengue virus were found: DENV-1, -2 and -3. No DENV-4 was found. We successfully isolated DENV-1, from which was sequenced the E gene for further analyses. Our analyses revealed that the current DENV-1 in Bhutan probably originated from India.

## Introduction

Dengue is one of the most common infectious diseases in tropical and sub-tropical regions of the world [1, 2]. The World Health Organization (WHO) estimates 50–100 million infections per year globally; however, other studies have suggested a much higher figure [2]. Southeast Asia and Western Pacific represent about 75% of the global dengue burden [3], causing a substantial economic cost in these regions [1].

Dengue virus (DENV), the etiological agent of dengue, is divided into four genetically and antigenically different serotypes, DENV-1 to 4 [4]. Although infection by a particular serotype is known to confer long-lasting homotypic immunity, circulating heterotypic antibodies are only able to provide transient cross-protective immunity often leading to severe forms of DF, dengue hemorrhagic fever (DHF) and dengue shock syndrome (DSS) [5]. Antibody dependent enhancement (ADE) and cross-reactive T-cell responses have been postulated to explain the possible mechanisms of disease enhancement [5, 6]. Other factors such as host immunity and viral genetics may contribute to severe forms of dengue fever (DF), dengue hemorrhagic fever (DHF) and dengue shock syndrome (DSS) [4, 5]. In recent years, DENV is increasingly being detected in newer geographical areas.

Dengue outbreaks are relatively new in Bhutan, a country that shares borders with India to the south and China to the north. The earliest documented dengue outbreak in the country occurred in 2004 andwas caused mainly by DENV-2 and 3 [7]. This was followed by sporadic dengue cases [8]. Although dengue is a reportable disease in Bhutan, it is believed to be inconsistently reported largely because diagnosis is clinically-based with rapid serological assays employed only in a few locations where laboratory diagnostic kits are available [9]. DENV molecular detection, isolation and other advanced testing have not yet been established in Bhutan. In addition, vector control efforts in Bhutan are mostly focused on malarial vector control, which is assumed to cover up for dengue as well. Dengue vector surveillances are in place where vectors were previously detected but no dengue-specific vector control measures have been implemented in the country [10].

Bhutan shares a 700 km border with India, which continues to report co-circulation of all 4 DENV serotypes with increasing frequency [11]. DENVs isolated in 2004–2006 from Bhutan during its first reported dengue outbreak are thought to have originated in India [7]. Similar transmission was reported in Nepal, a country with similar geographical features as Bhutan, gradually leading to endemicity [12]. Due to limited studies done in Bhutan, there is very little information regarding currently circulating DENV serotypes or their molecular and epidemiological characterization. In this study, we undertook laboratory confirmation of clinically suspected dengue patients from the southern part of Bhutan during 2013–2014 and elucidated the molecular epidemiology of DENV-1 in Bhutan.

## Materials and Methods

### Ethics statement

Samples were collected by the Public Health Laboratory in Bhutan as a part of routine diagnosis and surveillance; hence, no written consent was obtained from patients. The Ministry of Health (MOH), Bhutan, provided written permission for use of de-identified specimens and data for further evaluation. Approval of the study was provided by the Institutional Review Boards (IRBs) of Mahidol University, Thailand (COE. No. 2014/020/.1010), and Walter Reed Army Institute of Research (WRAIR), United States (WRAIR No. 2155).

### Specimen collection and study site

**Fig 1:**
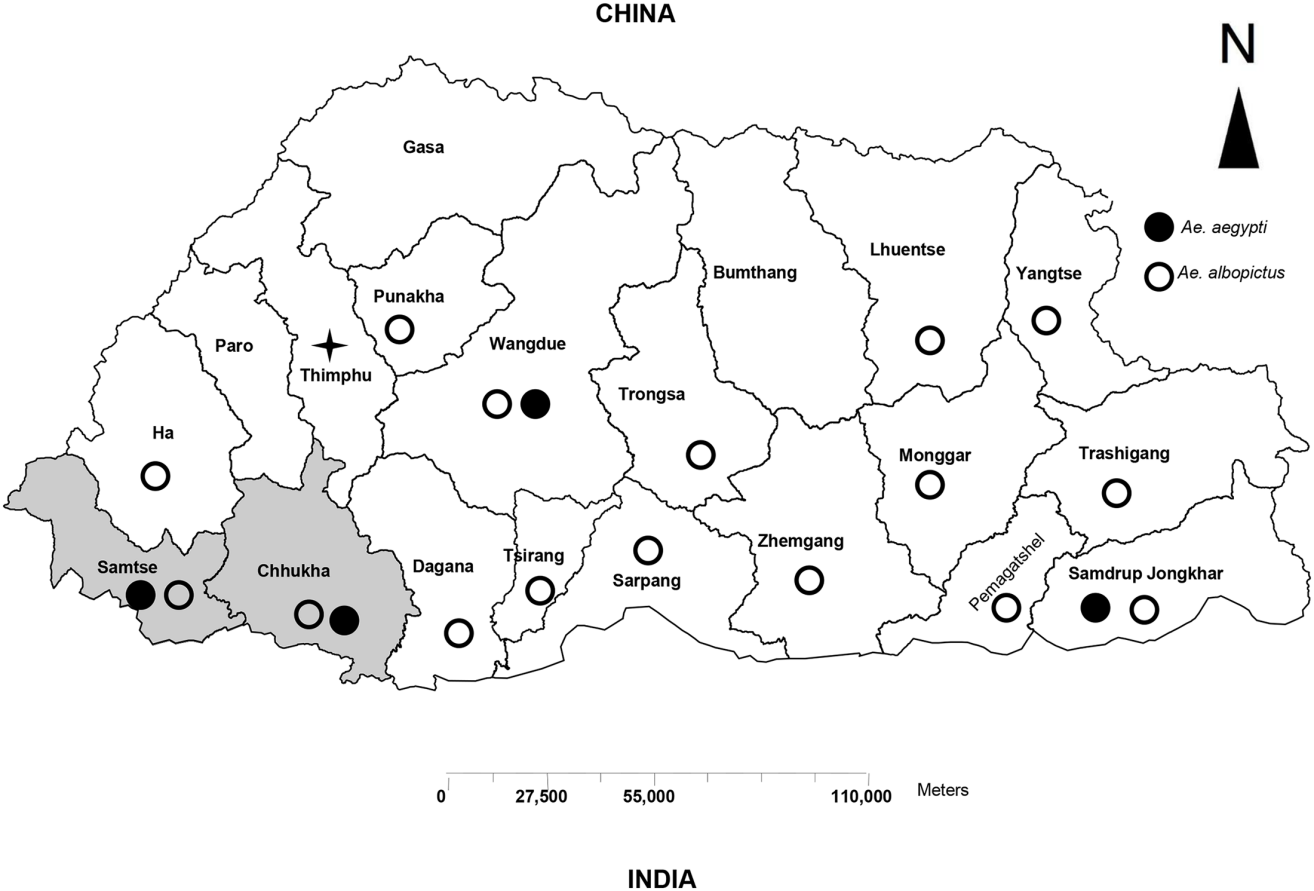
Map of Bhutan showing the distribution of dengue vectors in Bhutan. (*Source*: *Vector-borne Disease Control Program*, *Bhutan*). Shaded areas represent the sites of specimen collection for this study.

Acute blood specimens were collected over a period of two years, 2013–2014, from patients clinically suspected of having dengue. These patients had either visited the outpatient department (OPD) or were admitted to Samtse or Phuntsholing Hospitals in two southwest districts (Samtse and Chukha, respectively) of Bhutan (Fig 1). These districts are located at the foothills of the Himalayas where climate is sub-tropical. Both Samtse and Chukha share porous borders with India, where commerce and tourism are common. After the first outbreak of dengue in Phuntsholing town, Chukha district [7]; this area has continued to report dengue cases. Samtse district, which has similar climatic and ecological factors, was chosen as a site for this study along with Chukha. Other districts were not included in this study since they have had no reported cases of dengue infection. Both *Aedes* vectors (*Ae*. *Aegypti and Ae*. *Albopictus*) have been found in both Chukha and Samtse districts. These districts also have reasonable access to the Public Health Laboratory in the capital city, Thimphu, where specimens can be shipped for further evaluation

Clinically suspected dengue was defined as fever (oral, rectal or axillary temperature ≥38°C), or history of fever lasting 2 to 7 days of unknown origin with two or more of the following: headache, retro-orbital pain, myalgia, arthralgia, rash, hemorrhagic manifestation and leucopenia [13]. Patient demographic and clinical information was collected by attending clinicians. The clinical diagnosis of dengue by attending clinicians was not further categorized as to disease severity. Laboratory confirmation of dengue was carried out by DENV specific NS1 antigen and IgM ELISA, and nested RT-PCR.

### DENV NS1 antigen detection, IgM ELISA and nested RT-PCR

All acute serum specimens were tested for DENV infection by NS1 antigen detection, IgM ELISA and nested RT-PCR. Both dengue NS1 antigen and dengue IgM detection were carried out at the Public Health Laboratory, Bhutan using DENV Detect NS1 ELISA (InBios, Seattle, Washington) and Dengue Virus IgM ELISA (Calbiotech). Tests were performed according to manufacturer’s instructions. Acute serum specimens were also tested by nested RT-PCR at the Armed Forces Research Institute of Medical Sciences (AFRIMS) in Bangkok, Thailand. Viral RNA was extracted from 140 μl of sera using QIAamp viral RNA mini kit (QIAGEN, Germany) following the manufacturer’s instructions. Nested RT-PCR was performed using a method modified from Lanciotti et al as previously described [14, 15]. One step RT-PCR was carried out using AMV reverse transcriptase (Promega, Madison, WI, USA) and AmpliTaq DNA polymerse (Life Technologies, USA) in the first round PCR. Nested PCR was performed using AmpliTaq DNA polymerase in the second round PCR.

### Virus isolation, mosquito inoculation and IFA staining

All sera positive for DENV by nested RT-PCR were inoculated into freshly prepared mono-layers of C6/36 cells grown in Minimum Essential Medium (MEM, GIBCO) containing 10% heat inactivated fetal bovine serum (HIFBS), 1% Glutamine and 1% Penicillin and streptomycin. These cultures were maintained in maintenance medium (MM) containing RPMI with 5% HIFBS. A mock-infected C6/36 cell flask was included as a negative control. Cells underwent 3 passages and were observed for cytopathic effect (CPE). Identification of DENV serotypes was carried out by antigen capture ELISA as previously described [16, 17]. Molecular confirmation of the isolates was performed by extracting DENV RNA from the cell culture supernatant followed by nested RT-PCR. When cell-based DENV isolation was not possible, nested RT-PCR positive sera were inoculated into *Toxorhynchitis splendens* mosquitoes (0.3μl/ mosquito) as previously described [18]. Surviving mosquitoes were head squashed on microscopic slides and screened for flavivirus antigen by immunofluorescent antibody (IFA) staining. Virus isolates amplified from cell culture or mosquitoes were used in envelope (E) gene sequencing.

### Envelope gene sequencing and phylogenetic analysis

E gene of DENV was amplified using one-step RT-PCR amplification [19]. Overlapping fragments were amplified using AccessQuick RT-PCR System (Promega, Madison, WI, USA) with two sets of primers covering the entire E gene. Amplified products were purified prior to sequencing using QIAquick PCR purification kit (QIAGEN) following manufacturer’s instructions. Capillary-based Sanger sequencing was used to obtain E gene sequences (1,485 bp).

Base correction for the obtained sequences was performed using Sequencher 5.1. All new sequences were submitted to GenBank (accession numbers KP849860- KP849892). Maximum likelihood (ML) tree was constructed from 33 new Bhutan DENV-1 sequences along with sequences of 56 global DENV-1 and 3 vaccine strains downloaded from GenBank. The tree was constructed using MEGA v.6.0 (www.megasoftware.net) [20]; the Tamura Nei (TN93) model was chosen for nucleotide analysis. A sylvatic strain from Malaysia (accession no. AF425622) was used as the outgroup. Bootstrap value was obtained from 1000 replicates. Selection pressure among the Bhutan DENV-1 sequences was determined using the maximum likelihood approach of codon based test of selection available in Mega v.6.0. Percent identity of nucleotides and amino acids was calculated by Clustal W function available in MegAlign v.5.05 of DNASTAR package.

### Accession numbers

Accession numbers for E gene sequenced in this study: KP849860, KP849861, KP849862, KP849863, KP849864, KP849865, KP849866, KP849867, KP849868, KP849869, KP849870, KP849871, KP849872, KP849873, KP849874, KP849875, KP849876, KP849877, KP849878, KP849879, KP849880, KP849881, KP849882, KP849883, KP849884, KP849885, KP849886, KP849887, KP849888, KP849889, KP849890, KP849891 and KP849892.

### Statistical analysis

SPSS version 22 was used for statistical analysis. Independent T-test was used to compare means of various attributes. Frequencies/ percentage of clinical symptoms were compared using Pearson chi-square test. A probability value of p < 0.05 was considered statistically significant.

## Results

### DENV epidemiology

**Fig 2:**
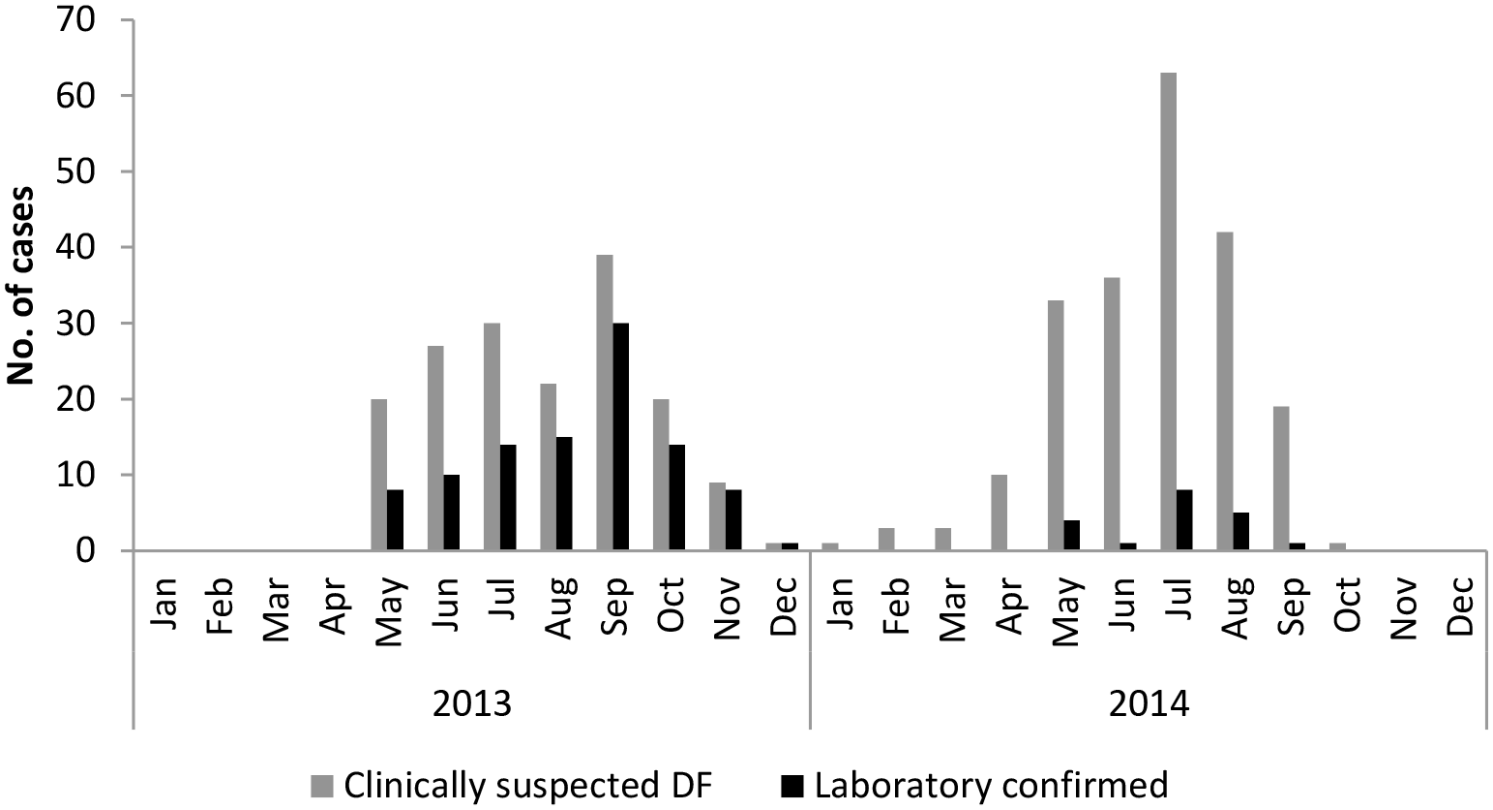
Epidemiological curve showing the distribution of cases in 2013 and 2014. The figure includes data collected from the two surveillance sites (Phuntsholing Hospital, Chukha district, and Samtse Hospital, Samtse district) of Bhutan.

A total of 379 acute sera from suspected dengue cases were collected at the district hospitals in Samtse and Chukha (Fig 1) during 2013 and 2014, of which 119 were laboratory confirmed for DENV infection. In both years, the number of suspected and laboratory confirmed cases peaked during the summer months (June-September), which also corresponds to the monsoon season in Bhutan (Fig 2). Clinically suspected and laboratory confirmed dengue cases during the summer months accounted for 278/379 (73%) and 83/119 (69.7%) respectively, for the entire two years. During the colder months (November to March), only 17/379 (4.5%) of all suspected dengue and 8/119 (6.7%) of laboratory confirmed dengue cases occurred. There seemed to be a remarkable difference in the proportion of laboratory confirmed dengue in 2013 and 2014. In 2013, 100/168 (59.5%) of suspected cases were confirmed to be dengue by laboratory methods, accounting for 84% of all laboratory confirmed dengue. Only 19/211 (9%) of suspected cases were laboratory confirmed in 2014.

Age of patients in this study ranged from 2 to 77 years but young adults, 19–35 years, accounted for the largest age group of suspected as well as laboratory confirmed cases; 178/379 (47%) and 68/119 (55.6%), respectively. Both genders were about equally affected (female to male ratio of 1:1.03). Mean age and days of illness (DOI) after onset of symptoms until blood collection were calculated separately for total suspected and laboratory confirmed cases (Table 1). We did not observe any differences among the two groups.

**Table 1:** Demography of suspected dengue cases along with mean days of illness (DOI) after onset of symptoms.

Year	Gender		Total no. of cases		Mean age			DOI		
	Male	Female	Suspected	Laboratory confirmed*	Suspected	Laboratory confirmed*	p-value	Suspected	Laboratory confirmed*	p-value
2013	95	73	168	100	28.9	29.2	0.85	3.35	3.2	0.7
2014	97	114	211	19	32.8	31.4	0.73	3.8	3.5	0.67
Total^†^	192	187	379	119	31.12	29.6	0.3	3.6	3.3	0.22

* By NS1 ELISA, IgM ELISA and/or RT-PCR

^†^ Total of 2013 and 2014

### Clinical features

Of the 379 suspected dengue patients, 364 visited the outpatient department (OPD) and the remaining 15 were either admitted or visited the emergency room. Clinical data was collected from all 379 patients. All patients had fever and most had features of DF such as headache, myalgia and joint pain (Table 2). Although we were unable to obtain complete information regarding the severity and classification of the disease, hemorrhagic manifestations characteristic of DHF were noted in some laboratory-confirmed dengue cases including petechiae, gastrointestinal bleeding (e.g., haematemesis and melena), and bleeding from the mucosa and/or other sites (Table 2).

**Table 2:** Clinical features recorded in suspected and laboratory confirmed dengue cases from southwest Bhutan, 2013–2014.

	Clinical symptoms (%)													
	Fever	Joint pain	Headache	Myalgia	Retro-orbital pain	Severe Backache	Rapid and weak pulse	Maculopapular rash	Hypotension	Mucosal bleeding	Haematemesis/ Melena	Petechiae	Positive torniquet test	Altered mental status
2013 (N = 168)	100	86.3	98.8	93.5	84.5	87.5	1.8	26.2	1.2	13.7	3.6	17.3	10.1	1.8
2014 (N = 211)	100	86.7	95.3	88.6	76.8	77.7	28.0	8.1	13.7	8.1	8.1	3.3	0.9	2.4
**P-value**	**-**	**1.00**	**0.07**	**0.07**	**0.07**	0.02*	<0.01*	<0.01*	<0.01*	**0.09**	**0.08**	<0.01*	<0.01*	**1.00**
Laboratory confirmed dengue (N = 119)	100	89.9	95.4	96.6	85.7	85.7	6.7	3.4	6.7	**15.1**	3.4	19.3	10.9	1.7
Suspected dengue ^†^ (N = 260)	100	85	95.4	88.1	77.7	80.4	20.8	10.4	8.8	**8.5**	7.3	5	2.3	2.3
**P-value**	**-**	**0.19**	**0.78**	<0.01*	**0.19**	**0.2**	<0.01*	<0.01*	**0.49**	**0.05**	**0.78**	<0.01*	<0.01*	**0.7**

N-total number of cases (denominator)

^†^Does not include laboratory confirmed dengue

### Association of DOI with NS1 and PCR test results

**Fig 3:**
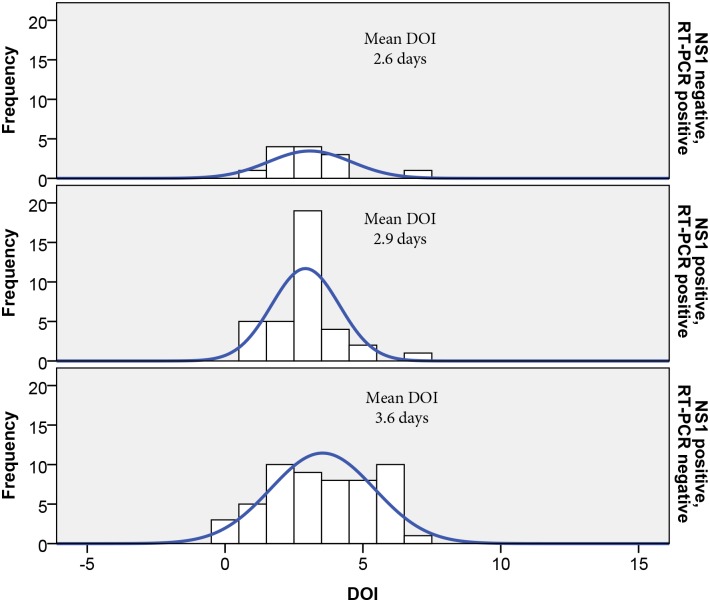
Mean DOI for NS1 and RT-PCR positive specimens. Mean DOI of (upper panel) NS1 negative, RT-PCR positive specimens; (middle panel) NS1 positive, RT-PCR positive; (lower panel) NS1 positive, RT-PCR negative.

A total of 97/379 (25.6%) specimens were positive by NS1 ELISA, 29/ 379 (7.6%) specimens were positive by IgM ELISA and 58/379 (15.3%) positive by nested RT-PCR. Combined, 119/ 379 (31.4%) specimens were positive by combination of these methods. The mean DOI was calculated separately for both NS1 and nested RT-PCR positive cases (Fig 3). Specimens positive for only NS1 had a DOI of 3.6 days, which was significantly longer than the DOI from samples positive for only nested RT-PCR (2.6 days, p<0.05). DOI for cases that were both NS1 and RT-PCR positive was 2.9 days. The DOI for IgM positive cases was 4.4 days.

### Serotyping and isolation

Nested RT-PCR was performed on all sera collected. Of 58 positive cases, 53 (91.4%) were DENV-1, 3 (5.2%) DENV-2 and 2 (3.4%) DENV-3; no DENV-4 was detected. Using cell culture and/or mosquito amplification, isolation of DENV was attempted on all 58 sera in order to obtain sufficient sequencing material. Unfortunately, we were unable to isolate any of the DENV-2 and DENV-3 viruses. Nevertheless, 33 viruses (all DENV-1) were successfully isolated and sequenced

### Phylogenetic and nucleotide sequence analyses

**Fig 4:**
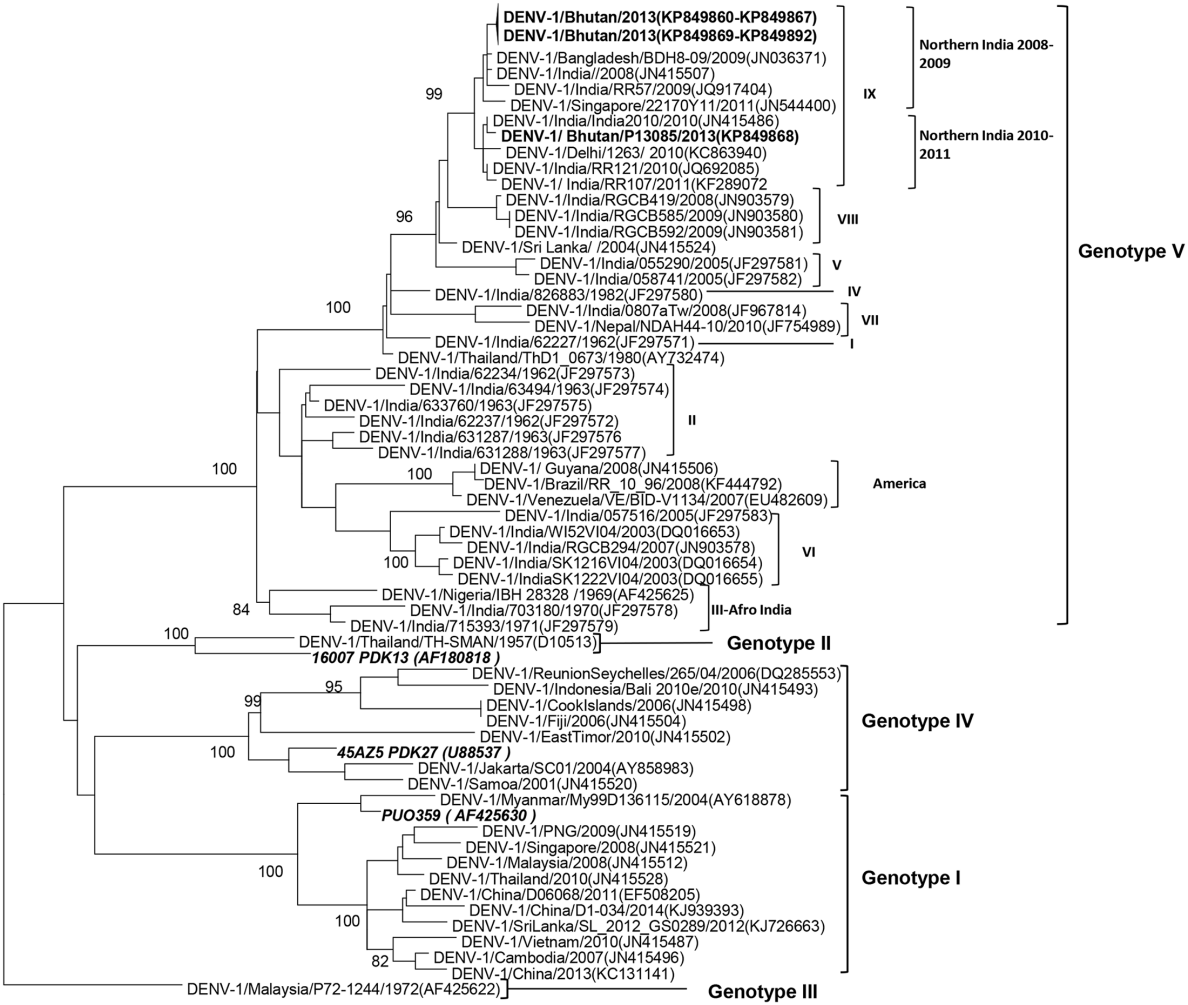
Phylogenetic tree. Maximum likelihood tree of 92 DENV-1 E gene sequences (1,485 bp) included 33 Bhutan strains (bold font) obtained from this study, and 56 global and 3 DENV-1 vaccine candidate sequences (italics) obtained from GenBank. A sylvatic strain from Malaysia (accession number AF425622) was used to root the tree. Bootstrap values are indicated at the major nodes. The sequences were named according to virus/country/strain/year of collection or isolation. GenBank accession numbers are shown in parentheses.

ML tree was generated using 92 E gene sequences (1,485 bp), including the 33 Bhutan DENV-1 strains reported here, 3 DENV-1 vaccine candidate strains and 56 global DENV-1 strains obtained from GenBank (Fig 4). All 33 Bhutan sequences group to genotype V, using the classification of Weaver et al [21], and were located in the same group as sequences from northern India, categorized as clade IX by Dash et al [22]. Within clade IX, 32 of the Bhutan DENV-1 E gene sequences (GenBank accession no. KP849860-7 and KP849869-92) grouped with the northern Indian sub-clade from 2008–2009, while 1 Bhutan DENV-1 sequence (GenBank accession no. KP849868) grouped with the northern Indian sub-clade from 2010–2011. None of the vaccine candidate strain sequences included in our phylogenetic tree fell within the same group as the Bhutan DENV-1.

Considering the diversity observed among the Bhutan DENV-1 E-gene sequences, we calculated the percentage of nucleotide and amino acid identity between the two observed sub-clades of Bhutanese specimens. We found 99.3–99.5% nucleotide identity and 99.6–99.8% amino acid identity. An I461V amino acid substitution was the only mutation found in the KP849868 sequence that differentiated it from the rest of the Bhutan sequences. This mutation was also found in an Indian sequence from 2010 (GenBank accession no. JN415486), but in no other sequence within the same clade. Selection pressure analysis using maximum likelihood approach showed that amino acids of Bhutan DENV-1 E region are under negative (purifying) selection with test statistics (d_S_-d_N_) = 2.7 bootstrapped with 1000 replicates.

## Discussion

This study demonstrated a higher number of cases during the summer season, especially affecting young adults, 19–35 years of age. Clinical presentation of these patients ranged from classical dengue fever to hemorrhagic manifestations. We established DENV-1 as the dominant of at least three DENV serotypes currently circulating in Bhutan. With a shift in predominant serotype from DENV-3 documented previously to DENV-1, dengue in Bhutan seems to follow a cyclical pattern as seen in other countries [23]. Phylogenetic characterization of DENV-1 revealed that they belong to genotype V [21], and were probably imported from India.

A distinct seasonality for DENV infection in the 2 southwest districts of Bhutan was observed. The number of cases was found to peak during the hot and humid monsoon season, probably due to increased habitats for mosquito breeding. Studies have shown a correlation between rainfall, temperature and humidity with serologically confirmed dengue [24, 25]. These climatic and ecological factors in combination with inadequate public services and ineffective vector control are known to contribute to dengue endemicity [24], all of which may be playing a role in Bhutan.

The majority of suspected and laboratory confirmed cases occurred in young adults aged 19–35 years. The same was observed in the previous study in Bhutan [7] where the mean age was found to range from 28 to 32 years of age. While the underlying pathogenesis leading to more symptomatic dengue in adults than in children is not clear, dengue as a disease of adults is supported by increasing DF notifications from many ageing countries [26, 27]. Ineffective vector control efforts have been proposed as a reason for causing shift in the age group from children to adults in countries like Singapore and Thailand [28, 29]. However; in the case of Bhutan, the majority of cases being adults suggest that there are still many dengue-naïve or dengue monotypic individuals among the adult population in Bhutan. Whether this mean age pattern changes in the future may depend on ongoing and future intensity of DENV transmission.

Considering the difference in sensitivity of NS1 ELISA and RT-PCR methods at various stages of illness [30], using both methods was useful in detecting cases that were positive by just one of the methods. As expected, results by each method varied and seemed to correlate with the number of days of illness after onset of symptoms. Interestingly, the percentage of NS1 ELISA-positive specimens (25.6%) was higher than the percentage of RT-PCR positive specimens (15.3%). It is unlikely that this is due to higher detection sensitivity by the NS1-ELISA method. One possible explanation is the preponderance of patients in this study seeking clinical care after several days of illness, favoring the detection of NS1, which circulates in the serum for longer periods than viral RNA [31]. The mean DOI among patients from whom virus genome could be detected was shorter than patients from whom only NS1 antigen could be detected (Fig 3). It is also possible that the commercial NS1 ELISA test, done in the laboratories in Bhutan and not confirmed at AFRIMS, provided a number of false positive results, accounting for a somewhat inflated positive percentage

A large numbers of specimens were negative for DENV by the laboratory methods used in this study, especially in 2014. One possible hypothesis could be that non-specific febrile illness, having fever with rash, can be caused by a number of other infectious diseases that may commonly be mistaken for dengue [32]. For example, an outbreak of chikungunya, which commonly manifests as fever and polyarthralgia, was recently reported in the same geographical location in Bhutan [33, 34]. Clinical manifestations that were observed at significantly higher frequencies in laboratory confirmed dengue as compared to the suspected dengue cases include petechiae and positive tourniquet test, symptoms that are specific to DENV infection. We have yet to confirm the etiology of the dengue-like illnesses in 2014. Other factors such as time of sampling, loss in viral titer as a result of long storage and transportation with several inevitable freeze- thaw cycles (caused by Bhutan’s poor road infrastructure and transportation networks), could have contributed to lesser DENV isolation and further sequencing in general.

The report of the first dengue outbreak in Bhutan suggested that DENV was imported from India, probably as early as 2004 [7]. DENV-3 was the predominant serotype in both Delhi (northern India) during 2003–2006 and Bhutan during 2004–2006 [7, 11, 22, 32]. Since 2006, DENV-1 prevalence has increased in India [22, 35, 36]. Similarly, in this study, DENV-1 was predominant in 2013–2014, further supporting the notion that dengue epidemiological patterns in Bhutan and India are closely linked with likely exchange of strains between the two countries.

Phylogenetic analysis of the 33 Bhutan DENV-1 isolates showed that all belong to DENV-1 genotype V, also known as the cosmopolitan or the American-African genotype because of its diversity [21, 22]. The Bhutan sequences were found to group with clade IX from northern India, the most recent Indian clade [22]. Inclusion of the Bhutan sequences from our study in the phylogenetic analysis resulted in the divergence of clade IX into two different sub-clades. One sub-clade consisted of sequences from 2008–2009 northern India, and the other of sequences from 2010–2011 northern India. Most of the Bhutan sequences grouped with the 2008–2009 northern Indian sequences, indicating that the 2013–2014 dengue viruses from Bhutan mainly originated from 2008–2009 northern India sub-clade viruses. Only one sequence (GenBank accession no. KP849868) grouped with the 2010–2011 northern India sub-clade. This virus was obtained from a specimen collected in August 2013 in Chukha district and was found to be associated with mild clinical symptoms (no hemorrhagic manifestations). It is difficult to ascertain whether the differences in these two sub-clades may have any effect on the severity of the disease since we had only one virus in the 2010–2011 northern India sub-clade. None of the vaccine candidate strains grouped in the same genotype as the Bhutan DENV-1 sequences. It is unknown whether these discrepancies would have any impact on the efficacy of possible future vaccinations in the region. DENV from Bhutan showed a strong negative (purifying) selection pressure which is usually observed in arboviruses [37]. This has been attributed to evolutionary constraints that make the viruses resistant to change, especially since their lifecycles include both vertebrate and invertebrate hosts.

There were several limitations to our study. Since we used pre-existing specimens and data, the available information was not complete, especially regarding disease severity, high risk occupational groups and certain clinical laboratory data such as blood counts. We also did not have access to convalescent sera and, therefore, could not confirm the non-acute dengue cases. Furthermore, this study was limited to only two hospitals in Bhutan so the data presented here may not necessarily represent the entire country.

Despite its relative isolation, Bhutan is seeing increased urbanization and travel, factors that have led to DENV of all serotypes co-circulating in many other countries [2, 38]. WHO has at least partially attributed outbreaks in Nepal and Bhutan to increasing global temperatures [10]. Although Bhutan is mostly situated at elevations of greater than a thousand meters above sea level, some areas of Chukha and Samtse districts are located as low as 100 meters above sea level, bearing climates that favor transmission of DENV and other tropical pathogens [39]. More importantly, these two districts share an unrestricted border with India which regularly reports dengue outbreaks of all serotypes [11], contributing to the DENV presence in Bhutan.

In this study, we report a shift in predominant DENV serotype in Bhutan in concert with prevailing patterns in neighboring India, along with epidemiological features of dengue in Bhutan. The southern part of Bhutan has likely become dengue endemic, hence enhanced and continuous dengue surveillance is required to generate more robust epidemiological data and to monitor for changes in the characteristics of circulating DENVs.

## Supporting Information

S1 Dataset Raw information of all specimens bearing age, gender, date of onset of illness, days of illness, signs and symptoms and laboratory results are presented. (XLSX)

S1 Checklist STROBE Checklist. (DOCX)
