# Supplementary material for: Epidemiological and Molecular Characterization of Dengue Virus Circulating in Bhutan, 2013-2014
Source: PLoS Negl Trop Dis. 2015 Aug 21;9(8):e0004010. doi: 10.1371/journal.pntd.0004010 (PMC4546418; doi:10.1371/journal.pntd.0004010)
Supplement: S1 Checklist — (DOCX) [file pntd.0004010.s002.docx]

STROBE Statement—checklist of items that should be included in reports of observational studies

|  | **Item No.** | **Recommendation** | **Page No.** | **Relevant text from manuscript** |
| --- | --- | --- | --- | --- |
| **Title and abstract** | 1 | (*a*) Indicate the study’s design with a commonly used term in the title or the abstract Epidemiological | 1 | Epidemiological characterization |
|  |  | (*b*) Provide in the abstract an informative and balanced summary of what was done and what was found | 1-2 |  |
| **Introduction** |  |  |  |  |
| Background/rationale | 2 | Explain the scientific background and rationale for the investigation being reported | 2-4 |  |
| Objectives | 3 | State specific objectives, including any prespecified hypotheses | 4 | undertook laboratory confirmation of clinically suspected dengue |
| **Methods** |  |  |  |  |
| Study design | 4 | Present key elements of study design early in the paper | 4 | Specimen collection and study sites |
| Setting | 5 | Describe the setting, locations, and relevant dates, including periods of recruitment, exposure,  follow-up, and data collection | 4 | Both Samtse and Chukha share porous borders with India. Acute blood specimens were collected over a period of 2two years, 2013-2014, from patients clinically suspected of having dengue |
| Participants | 6 | (*a*) *Cohort study*—Give the eligibility criteria, and the sources and methods of selection of participants. Describe methods of follow-up  *Case-control study*—Give the eligibility criteria, and the sources and methods of case ascertainment and control selection. Give the rationale for the choice of cases and controls *Cross-sectional study*—Give the eligibility criteria, and the sources and methods of selection of  participants | 4 | patients had either visited the outpatient department (OPD) or were admitted to Samtse or Phuntsholing Hospitals in two southwest districts. Clinically Suspected dengue was defined as fever (oral, rectal or axillary temperature >38ºC), or history of fever lasting 2 to 7 days of unknown origin with two or more of the following: headache, retro-orbital pain, myalgia, arthralgia, rash, hemorrhagic manifestation and leucopenia |
|  |  | (*b*) *Cohort study*—For matched studies, give matching criteria and number of exposed and unexposed  *Case-control study*—For matched studies, give matching criteria and the number of controls per  case |  |  |
| Variables | 7 | Clearly define all outcomes, exposures, predictors, potential confounders, and effect modifiers.  Give diagnostic criteria, if applicable | 5 | ]. Patient demographic and clinical information was collected by attending clinicians. The clinical diagnosis of dengue by attending clinicians was not further categorized as to disease severity. Laboratory confirmation of dengue was carried out by DENV specific NS1 antigen and IgM ELISA, and nested RT-PCR. |
| Data sources/  measurement | 8* | For each variable of interest, give sources of data and details of methods of assessment  (measurement). Describe comparability of assessment methods if there is more than one group | 5 | All outcomes are limited to initial clinical diagnosis and laboratory findings |
| Bias | 9 | Describe any efforts to address potential sources of bias |  | This was an observational study |
| Study size | 10 | Explain how the study size was arrived at |  | The size of the study was established simply by the number of patients who sought clinical care at the sites defined above. |
| Continued on next page |  |  |  |  |

Quantitative variables Statistical methods

**Results**

1. Explain how quantitative variables were handled in the analyses. If applicable, describe which groupings were chosen and why: **There were no groupings other than positive/negative based on laboratory results**
2. (*a*) Describe all statistical methods, including those used to control for confounding: **all analytical methods are described in the paper**
   1. Describe any methods used to examine subgroups and interactions: **none was used**
   2. Explain how missing data were addressed: **there was no missing data**
   3. *Cohort study*—If applicable, explain how loss to follow-up was addressed

*Case-control study*—If applicable, explain how matching of cases and controls was addressed *Cross-sectional study*—If applicable, describe analytical methods taking account of sampling strategy

- 1. Describe any sensitivity analyses: **None was done**

Participants 13* (a) Report numbers of individuals at each stage of study—eg numbers potentially eligible, examined for eligibility, confirmed eligible, included in the study, completing follow-up, and analysed: **all samples were analyzed in one stage and deemed eligible.**

- - 1. Give reasons for non-participation at each stage: **None**
    2. Consider use of a flow diagram. **None necessary**

Descriptive data 14* (a) Give characteristics of study participants (eg demographic, clinical, social) and information on exposures and potential confounders: **this was not a prospective cohort study**

1. Indicate number of participants with missing data for each variable of interest. **none**
2. *Cohort study*—Summarise follow-up time (eg, average and total amount) Outcome data 15* *Cohort study*—Report numbers of outcome events or summary measures over time

*Case-control study—*Report numbers in each exposure category, or summary measures of exposure

*Cross-sectional study—*Report numbers of outcome events or summary measures

Main results 16 (*a*) Give unadjusted estimates and, if applicable, confounder-adjusted estimates and their precision (eg, 95% confidence interval). Make clear which confounders were adjusted for and why they were included

1. Report category boundaries when continuous variables were categorized
2. If relevant, consider translating estimates of relative risk into absolute risk for a meaningful time period

Continued on next page

| Other analyses | 17 | Report other analyses done—eg analyses of subgroups and interactions, and sensitivity analyses |
| --- | --- | --- |
| **Discussion** | | |
| Key results | 18 | Summarise key results with reference to study objectives: **This was a descriptive study. DENV-1 was predominant in Bhutan during the 2013-14 outbreak.** |
| Limitations | 19 | Discuss limitations of the study, taking into account sources of potential bias or imprecision. Discuss  both direction and magnitude of any potential bias: **Major limitation was the limited number of patients and limited number of sites** |
| Interpretation | 20 | Give a cautious overall interpretation of results considering objectives, limitations, multiplicity of  analyses, results from similar studies, and other relevant evidence: **These results should provide sufficient evidence that dengue is endemic in Bhutan and that public health policies should be drafted to track this virus better** |
| Generalisability | 21 | Discuss the generalisability (external validity) of the study results: **Dengue virus may be endemic in Bhutan.** |
| **Other information** | | |
| Funding | 22 | Give the source of funding and the role of the funders for the present study and, if applicable, for the  original study on which the present article is based: **Funding was obtained from AFHSC-GEIS. AFHSC-GEIS did not participate in design of this study, the analyses of the data or in the preparation of this manuscript.** |

*Give information separately for cases and controls in case-control studies and, if applicable, for exposed and unexposed groups in cohort and cross-sectional studies.

**Note:** An Explanation and Elaboration article discusses each checklist item and gives methodological background and published examples of transparent reporting. The STROBE checklist is best used in conjunction with this article (freely available on the Web sites of PLoS Medicine at [http://www.plosmedicine.org/,](http://www.plosmedicine.org/) Annals of Internal Medicine at [http://www.annals.org/,](http://www.annals.org/) and Epidemiology at [http://www.epidem.com/).](http://www.epidem.com/)) Information on the STROBE Initiative is available at [www.strobe-statement.org.](http://www.strobe-statement.org/)
